# Development of Biomedical Polymer-Silicate Nanocomposites: A Materials Science Perspective

**DOI:** 10.3390/ma3052986

**Published:** 2010-04-28

**Authors:** Chia-Jung Wu, Akhilesh K. Gaharwar, Patrick J. Schexnailder, Gudrun Schmidt

**Affiliations:** Weldon School of Biomedical Engineering, Purdue University, West Lafayette, IN 47907, USA; E-Mails: wu97@purdue.edu (C.J.W.); agaharwa@purdue.edu (A.K.G.); pschexna@purdue.edu (P.J.S.)

**Keywords:** nanocomposite, polymer, silicates, clay, biopolymer, structure, mechanical properties, bio-technology, bioactive, biomedical

## Abstract

Biomedical polymer-silicate nanocomposites have potential to become critically important to the development of biomedical applications, ranging from diagnostic and therapeutic devices, tissue regeneration and drug delivery matrixes to various bio-technologies that are inspired by biology but have only indirect biomedical relation. The fundamental understanding of polymer-nanoparticle interactions is absolutely necessary to control structure-property relationships of materials that need to work within the chemical, physical and biological constraints required by an application. This review summarizes the most recent published strategies to design and develop polymer-silicate nanocomposites (including clay based silicate nanoparticles and bioactive glass nanoparticles) for a variety of biomedical applications. Emerging trends in bio-technological and biomedical nanocomposites are highlighted and potential new fields of applications are examined.

## 1. Introduction

A fundamental understanding of polymer-nanoparticle interactions is necessary to control the structure-property relationships of polymer nanocomposites that need to work within the chemical, physical and biological constraints required by a biomedical application. Polymers are widely used biomaterials, as the range of their chemical and physical properties can be varied [1,2,3]. With an understanding of polymer molecular structure in the 1920’s, the field of polymer science was born [4]. Although this discipline began with the characterization of biological polymers, the field developed further through synthetic polymers in the 1950’s [5], and later branched into many different directions, among them biomaterials [6,7,8,9,10], polymer nanocomposites [11,12,13], and polymer nanocomposite biomaterials [14,15]. Today, the interdisciplinary nature of the polymer nanocomposite biomaterials field brings together researchers from polymer science, biology, materials and biomedical engineering, chemistry and physics. Such collaborative work often leads to the generation and use of new terminology and definitions that are used across the individual disciplines. For example, recent literature suggests that the 1999 traditional definition of biomaterials [16] has changed with time as these materials find use in a variety of medical and nonmedical technology that is inspired by biology [6,16]. Thus it becomes more difficult to classify between polymer nanocomposite biomaterials that are developed for biomedical devices and polymer nanocomposite biomaterials that are used for nonmedical bio-technological purposes (e.g., renewable resources). Here we will review polymer nanocomposite materials that have potential to be used as biomaterials in the biomedical field where they interface with tissues or tissue components [16].

A large body of literature covers polymeric biomaterials that are developed to substitute and repair biological tissues [17,18], Among these, several new approaches attempt to design self-assembled and smart nanocomposite biomaterials that respond to external stimuli such as optic, temperature, mechanic, electric and magnetic fields [9,19,20]. The sensitivity of these materials to external stimuli is very important in the design of smart implants and drug delivery systems as well as new bio-technologies including biosensors, actuators, *in vitro* diagnostics, cell culture matrixes, contrast agents and bioassays [19,20].

The optimization of complex polymer bio-nanocomposite materials for emerging technologies such as scaffolding, tissue regeneration and controlled drug delivery creates new hopes for the faster and better treatment of diseases. One of the most promising, but also most difficult, challenges that researchers face is the creation of polymeric nanocomposites that have not only superior performance and mechanical properties but also acceptable biological function. For example, a polymer nanocomposite that appears to be non-cytotoxic, *in vitro*, is not necessarily biocompatible, *in vivo* [1]. Despite much success in controlling either the chemical and biological properties individually, the physical properties, specifically the mechanical properties, of biological tissues are difficult to replicate. The mechanical performance of complex biological tissue surpasses most of the engineered polymer materials, and research is needed to determine what makes biological tissue so robust. Several research groups investigated the mechanical properties of selected biological tissues and concluded that a polymer nanocomposite structure is often responsible for the mechanical properties, and that nanoscale hard inclusions are frequently dispersed within a softer biopolymer matrix (e.g., bone) [21,22,23,24]. Unique combinations of hard and soft components found in biological tissues inspired materials researchers to design and develop polymer nanocomposites with improved mechanical properties [13,22,25,26,27,28]. Several of these polymer nanocomposites have potential to be used as biomaterials.

Some approaches to improve materials performance include the synergistic combination of chemical, physical and biological properties. If successful, polymer nanocomposites can combine the most suitable characteristics of nanoparticles and nanostructures with those of the polymer matrix. By tuning multiple parameters at the same time, a broad spectrum of functionalities are being developed that can be used for engineering new materials for specific biomedical products [17].

**Figure 1:**
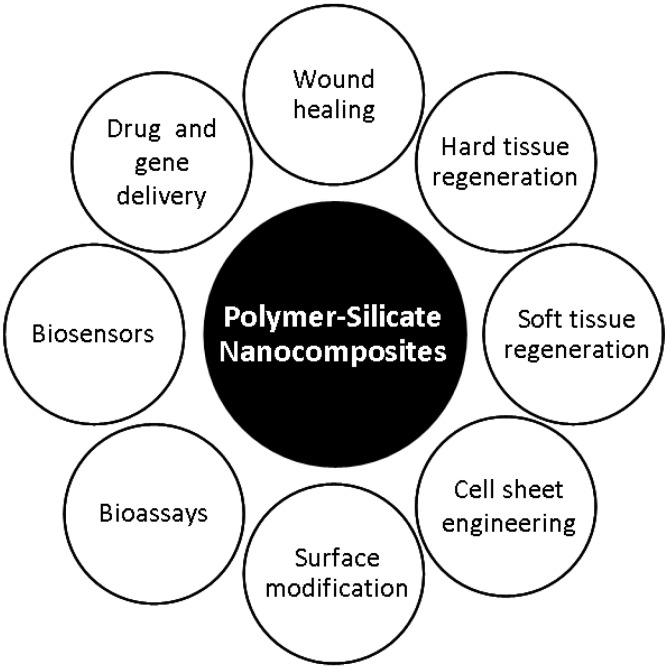
Polymer-silicate nanocomposites have been developed to address a multitude of biomedical applications.

This review summarizes the most recent strategies to design and develop polymeric nanocomposite materials for diverse biomedical applications. A variety of polymeric bio-nanocomposite materials are generated by the combination of inorganic nanoparticles with polymers of synthetic or natural origin (Figure 1). Nanocomposites made from biomedical polymers and silicate nanoparticles are reviewed while highlighting their potential and shortcomings in the biomedical and bio-technological arenas.

Overall, this literature review will show that only some of the material properties can be tailored to increase specific functionality and to optimize performance in a biological environment. Fundamental studies of the structures and properties as well as molecular analysis are highlighted, as basic design principles become increasingly important to the optimization and formulation of already existing biomaterials. The development of polymer nanocomposites for other applications such as biomedical nanotechnology is covered together with emerging new trends in bio-technological polymer nanocomposites that may have biomedical relevance in the future. Finally, future trends and challenges are summarized that guide polymer nanocomposite biomaterial design.

## 2. Biomedical Polymers Reinforced with Clay Based Silicate Nanoparticles

Silicate nanoparticles have been extensively used to improve the mechanical properties of synthetic and natural polymers (Table 1). The resulting polymer nanocomposites often show significant improvements in structure, modulus, strength and toughness, all properties that cannot be achieved by using the polymer alone [11,12,13,29]. Thus, polymers commonly used for biomedical applications have been reinforced by the addition of silicate nanoparticles. However, only few reports focus on polymer nanocomposites that may eventually achieve biomedical relevance [14]. Some of the challenges to consider when developing silicate reinforced polymers for biomedical applications include the potential accumulation of non-degradable silicate nanoparticles *in vivo* and long-term biocompatibility issues with these materials, as non-cytotoxic does not necessarily mean *in vivo* biocompatibility.

**Table 1:** Some biomedical polymers reinforced with Montmorillonite (MMT clay) and Cloisite (modified MMT clay) nanoparticles.

Nanoparticles	Polymer	Experimental observations	Ref
MMT	PLG	Toughness and elongation of the nanocomposites enhanced due to addition of nanoparticles. Physical cross-linking between polymer and nanoparticles triggered a toughening mechanism via multiple crazing and shear yielding	[30]
MMT	PLLA	Increase in tensile modulus observed with addition of MMT. Enhanced surface interaction between nanoparticles and polymer decreased polymer crystallinity and promoted degradation of the nanocomposite	[31]
MMT	PLLA	MMT improved structural integrity of the nanocomposites	[33,34]
MMT	PLA	MMT improved compression properties and hydrophilicity of the polymeric matrix	[35]
MMT	PLLA	Higher amounts of MMT and fully exfoliated structures gave rise to stiffer materials. Addition of MMT suppressed polymer crystallization due to enhanced surface interactions	[36]
MMT	Gelatin-chitosan	Lower degradation rate and enhanced cell adhesion observed after addition of MMT to the polymer blend	[37]
Cloisite	Ethylene vinyl acetate	10% clay concentration produced materials with the higher moduli and enhanced cell proliferation	[38]
Cloisite	Polyurethanes	Nanocomposites had a 5 fold lower permeability towards water vapor and enhanced mechanical properties	[40,41]

While much of the literature in this area is focused on improving mechanical properties, biological constraints are not always adequately addressed. For example, poly(lactic–co-glycolide) (PLG) is a biocompatible and biodegradable, but brittle, polymer that has been considered for sutures, resorbable meshes and controlled drug release [2,30]. The addition of small amounts of surface modified clay nanoparticles (natural Montmorillonite clay, MMT = layered silicate) to this polymer can improve its toughness and elongation during tensile tests from 7% for the neat polymer to 210% for the polymer nanocomposite [30]. The authors attributed this reinforcement and toughness to the physical cross-linking between polymer chains and MMT silicate nanoparticles. These physical cross-links increase the fracture strength of the polymer and trigger a toughening mechanism via multiple crazing and shear yielding [30].

In another study, Lee *et al*. have used silicate clay nanoparticles (MMT) to improve the mechanical properties of model scaffolds made of poly L-lactic acid (PLLA) [31]. The resulting polymer nanocomposite scaffolds had a 40% increase in tensile modulus when compared to pristine PLLA scaffolds. Because the addition of MMT nanoparticles decreased the polymer crystallinity, the resulting nanocomposite scaffolds showed faster biodegradation than the neat polymer [31,32]. The authors concluded that the mechanical strength and biodegradation of the PLLA nanocomposites could be tailored by the addition of layered silicate nanoparticles. In related studies by the same and other authors, the biodegradation morphology of silicate-PLLA nanocomposites was reported in more detail [33,34], and the scaffolds obtained by fiber-spinning exhibited improved structural integrity during biodegradation of the polymer. Fiber scaffolds have potential to be further developed in bone-cartilage and bone-ligament interfacial applications. This is due to the porous nature of fiber scaffolds promoting cellular infiltration, along with strong mechanical properties of fibrous materials. Unfortunately, neither the end fate nor the degradation of the natural Montmorillonite clay (MMT) within the PLLA scaffolds was addressed, and this remains a critical issue, especially since the authors envision these materials to be used as degradable tissue engineering scaffolds. As shown in the next sections of this review, the presence of silicate nanoparticles may support the formation and repair of bone; thus PLLA-clay nanocomposites might have significant biomedical potential.

Ozkoc *et al*. fabricated porous PLA-MMT nanocomposites (PLA: poly lactic acid) using microcompounding and polymer/particle leaching [35]. Addition of MMT improved the compression properties of the polymer nanocomposites to be close to those of cancellous bone. The hydrophilicity of the polymer nanocomposite surfaces directly affected the cell adhesion. For example, addition of 3% MMT reduced the water contact angle from 60.7° to 31.4°. This is due to a decrease in interfacial tension between polymer and water, making the PLA surface more hydrophilic.

Similarly to Lee *et al*., Krikorian *et al*. reported significant improvement in mechanical properties of PLLA due to the addition of MMT [36]. Higher amounts of MMT and exfoliated structures gave rise to stiffer materials compared to microphase separated or intercalated composites. Exfoliated nanoparticles suppressed polymer crystallization due to enhanced surface interactions. Moreover, an increase in silicate concentration and exfoliation resulted in stiffer and transparent PLLA-MMT nanocomposites. The authors did not report on any potential biomedical applications their materials might have.

Zhuang *et al*. showed that the intercalated structure of MMT-gelatin-chitosan has a lower degradation rate when compared to a gelatin-chitosan scaffold, and that the degradation rate can be altered by changing the MMT concentration [37]. Enhanced cell adhesion and proliferation on the MMT-gelatin-chitosan nanocomposite film was observed. Chitosan may also be utilized in the production of glycosaminoglycans, which can aid in cartilage integration.

Other nanocomposites made from ethylene vinyl acetate and natural Cloisite clay were investigated by Lewkowitz-Shpuntoff *et al.* [38]. These authors reported on the clay dependent mechanical properties and the adhesion and growth of human dermal fibroblasts on the polymer nanocomposite surfaces. The mechanical testing data suggested that a 10% clay concentration produced materials with the highest moduli. In a similar way, cell growth was found to be highest on the surfaces of polymer nanocomposites containing 10% clay. Fibroblast cells cultured on substrates with higher clay content had poor growth curves, and misshaped actin fibers. By adsorption of iron onto the Cloisite clay, the resulting polymer nanocomposites became magnetic which enhanced proliferation of MC3T3 osteoblast cells on the materials surface. Osteoblast cell proliferation was maximized by culture on electrospun aligned fibers in a constant magnetic field [38]. Such an ability to align different cell types provides for the further development of this system for tendon or bone repair.

The barrier properties of polymer nanocomposites can be used in sealed medical devices to isolate power supplies and microelectronics, such as pacemakers, from the wet environment of the body. For example, biomedical polymers used in the development of pacemakers, implantable artificial hearts and left ventricular assist devices must exhibit not only suitable mechanical properties but be non-thrombogenic and have low calcification and permeation properties. Polyurethanes are one of the classic biomaterials frequently used for these purposes because they combine superior flexural performance with good blood compatibility. Unfortunately, polyurethanes are relative permeable to water, air and water vapor, which may cause failure of the microelectronics they are supposed to seal [39]. In order to reduce permeability and maintain the desirable biocompatibility and mechanical properties, organic modified silicate nanoparticles (modified MMT = Cloisite clay) were added to biomedical polyurethanes [40]. The resulting polyurethane-silicate nanocomposites had a 5 fold lower permeability towards water vapor when compared to the pristine polymer. In addition, the mechanical properties of the polymer nanocomposite were also significantly enhanced [40,41].

## 3. Polymer Silicate Nanocomposite Hydrogels with Biomedical Potential

In addition to bulk materials, silicate nanoparticles (from layered clay) can be used to significantly improve the mechanical properties of polymer hydrogels (Table 2). Hydrogels are of great interest in the biomedical engineering field because of their similarity to soft tissues. However, the low mechanical strength of hydrogels often limits their practical applications. In order to improve the mechanical strength of hydrogels with biomedical potential, several authors have used silicate nanoparticles (clay) as either fillers or cross-linkers to strengthen the polymer network [42,43,44]. A silicate often used as a physical, or covalent, cross-linker to the polymer is Laponite, which is comprised of synthetic and charged silicate nanoparticles. Advantages of using synthetic Laponite over the previously mentioned natural Montmorillonite include single layer dispersions of nanoparticles, high purity, gelation properties and its previous use in pharmaceutical and cosmetic applications. Polymers frequently used for synthesizing silicate cross-linked polymer hydrogels are poly(acryl amide) and poly(ethylene oxide) (PEO) [42,43,44]. Stimuli sensitive poly(N-isopropylacrylamide) (PNIPAM) hydrogels are already attractive biomaterials used for drug delivery, bioseparation devices and culture dishes for cell sheet engineering. The lower critical solution temperature and the thermo sensitive coil to globule transition of PNIPAM are suitable for use in a variety of biomedical applications.

**Table 2:** Polymer-Laponite nanocomposite hydrogels with biomedical potential.

Nanoparticles	Polymer	Experimental observations	Ref
Laponite	PNIPAM	Ultrahigh elongation with near-complete recovery, rapid de-swelling responses to temperature changes and large equilibrium swellings were observed due to addition of Laponite to the polymeric matrix.	[42,46,47,48]
Laponite	PNIPAM	Cell sheet easily detached by changing temperature.	[43]
Laponite	PEO	Cells cultured on the surfaces of PEO-Laponite gels attached and proliferated easily.	[53,54]

**Figure 2:**
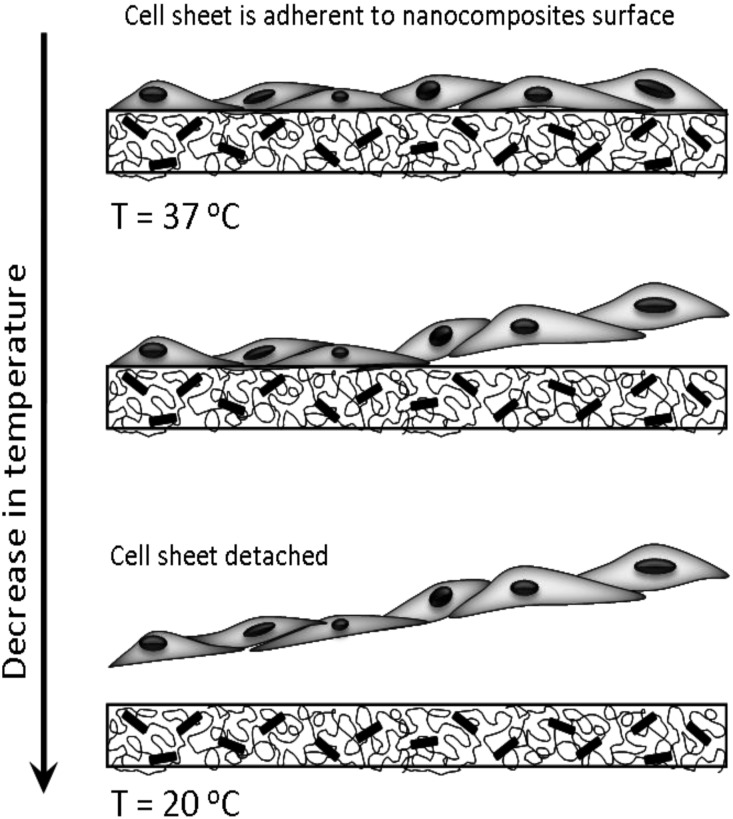
Cell sheets can be isolated by culturing cells on temperature responsive polymer nanocomposite materials. After reaching confluency, a cell sheet is detached by decreasing the temperature. When the temperature drops below the lower critical solution temperature, the change in polymer conformation causes cells to detach [43].

Although the exact molecular interactions between polymer and silicate nanoparticles are still not clear, cross-linking PNIPAM to Laponite requires monomer polymerization to be started from the silicate nanoparticles [42,45]. The resulting hydrogels have structurally homogeneous distributions of silicate cross-linkers that allow for dissipation of stresses during mechanical deformation. Mechanical testing data of these PNIPAM-Laponite hydrogels suggest ultrahigh elongation with near-complete recovery, rapid de-swelling responses to temperature changes and large equilibrium swellings [42,46]. More than 1000% elongations were observed with tensile moduli ranging from KPa to MPa [46]. Both, the tensile and compressive moduli were dependent on silicate concentration [47,48]. The compressive moduli could be further improved by introducing additional covalent cross-linkers [49]. This polymer nanocomposite approach has been validated with different combinations of silicate nanoparticles and polymers, however biomedical relevance was mentioned by few groups [43]. The authors mention in the introduction of their paper that their highly extensible hydrogels exhibit good blood compatibility and no inflammation when tested in an animal model [43]. The first PNIPAM-Laponite nanocomposite hydrogels were first developed by Haraguchi *et al.* [42], who later studied cell cultivation and cell sheet detachment on the hydrogel surfaces [43]. Cell adhesion and proliferation of human hepatoma cells, dermal fibroblasts and umbilical vein endothelial cells were found to be strongly dependent on the silicate concentration. The stimuli-responsiveness of PNIPAM nanocomposites to temperature was used to spontaneously separate confluent cell sheets from the hydrogel surfaces, thus offering new strategies for cell sheet engineering (Figure 2) [43]. We categorize this new PNIPAM nanocomposite to be a biomaterial or biomedical material as it interfaces directly with a living cell sheet [16].

Other polymer nanocomposite hydrogels that have potential biomedical relevance are made from PEO that is physically cross-linked to Laponite [50]. PEOs are among the most researched water-soluble synthetic polymers that have attracted much interest in the biomedical field [51]. The ability to prevent protein denaturation and its biocompatibility makes PEO-modified materials suitable for supporting cell growth. When PEO is mixed with Laponite nanoparticles, the polymer readily adsorbs and desorbs from the nanoparticle surfaces forming mechanically strong, self-healing and injectable hydrogels. Although published work on these type of hydrogels goes back more than a decade [52]. the molecular interactions between polymers and silicate nanoparticles are not clear. Therefore, PEO-Laponite hydrogels are one of the most studied model systems for investigating fundamental polymer-clay interactions and shear-orientations. Biomedical relevance has been suggested only recently when cell growth studies showed that murine fibroblast cells cultured on the surfaces of PEO-Laponite gels attach and proliferate easily [53,54]. Inclusion of chitosan to PEO-Laponite hydrogels improved cell adhesion and spreading, while maintaining mechanical strength, injectability and self-healing, all of which are dominated by the synthetic polymer and Laponite components. Thus small amounts of chitosan add advantageous properties without hampering the mechanical strength of the nanocomposite hydrogel [54].

## 4. Polymer Layered Silicate Nanocomposite Developments for Drug Delivery Applications

**Figure 3:**
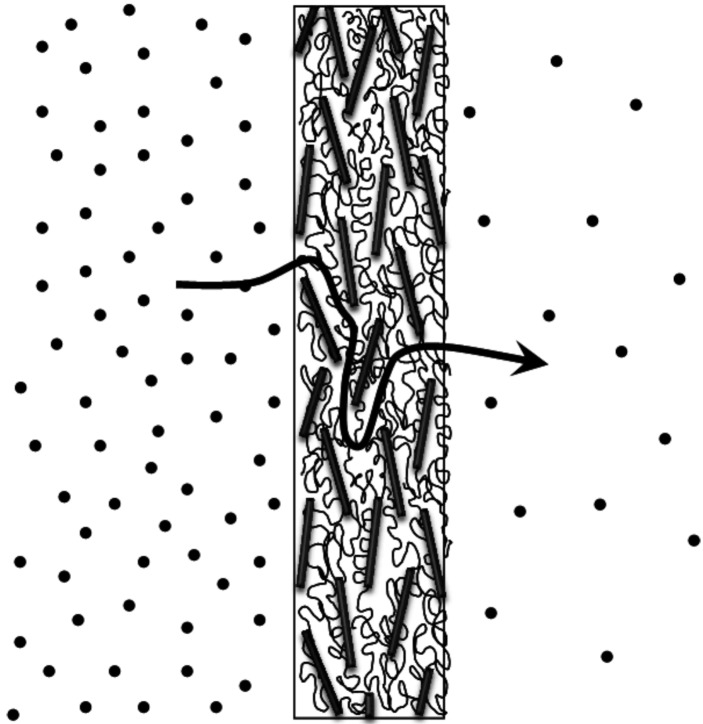
Silicate based polymer nanocomposite materials exhibit reliable barrier properties due to the tortuous path small molecules must travel to pass through the material, making these materials useful in sustained drug delivery applications.

One of the problems biomaterials for drug delivery need to overcome is the burst release of encapsulated or entrapped drugs. By controlling the release kinetics of drugs, one can not only optimize the therapeutic effects of the drug, but also influence their biological activity. Silicate based polymer nanocomposites demonstrate good barrier properties due to the tortuous diffusion pathways that small molecules must travel in order to clear the material (Figure 3) [55]. This property can be used towards the development of sustained drug release applications. Model drugs have been loaded into nanocomposites made of various combinations of biomedical polymers and clays (Table 3). For example, organic modified silicate nanoparticles (Cloisite clay) were added to poly(ethylene-co-vinyl acetate) to study the release kinetics of dexamethasone [56]. The authors discovered that the increase of silicate nanoparticle concentration resulted in higher mechanical strength of the polymer nanocomposite and a sustained release of dexamethasone. The drug release kinetics was suggested to be dependent on the aspect ratio and degree of dispersion of the silicate nanoparticle [56].

Injectable delivery of drugs can also be achieved with polymer nanocomposites by using polymers that respond to external stimuli, such as temperature. In one example, Wu *et al*. reported on the temperature dependent sol-gel transitions in thermosensitive nanocomposite hydrogels made from Laponite nanoparticles and Pluronic type polymers [57]. Pluronics are thermosensitive triblock copolymers composed of poly(ethylene oxide)- poly(propylene oxide)- poly(ethylene oxide) (PEOx-PPOy-PEOz), and are important in injectable applications. The fast dissolution properties of pure Pluronic hydrogels hinder their use for long-term drug release applications. However, the addition of silicate nanoparticles was found to shift the Pluronic phase transition temperature and to enhance the dissolution resistant properties of the hydrogels. As a consequence, the release of a macromolecular model drug, albumin, could be significantly lengthened [57].

**Table 3:** Polymer layered silicate nanocomposite developments for drug delivery applications.

Nanoparticles	Polymer	Experimental observations	Ref
Cloisite	Poly(ethylene-co-vinyl acetate)	Addition of nanoparticle resulted in slower release of dexamethasone. Moreover, release kinetics were dependent on the aspect ratio and degree of dispersion of the nanoparticle	[56]
Laponite	Pluronic	A temperature dependent sol-gel transition was observed in the nanocomposites. Laponite enhanced the dissolution resistant properties of the hydrogels and release of entrapped macromolecular drug was slowed down	[57]
Bentonite	Acrylic acid-PEG methyl ether acrylate	Elution kinetics strongly depended on the interactions between the surface charges of the clay and the drug	[58]
Laponite	PEO-polyamide	Molecular interactions between Laponite and drug resulted in sustained release profiles	[59]

A study by Lee and Chen described the delivery of model drugs from hydrogels made of acrylic acid-poly(ethylene glycol) methyl ether acrylate and natural Bentonite clay nanoparticles [58]. These authors found that the elution kinetics are strongly dependent on the interactions between the surface charges of the clay and the drug. Vitamin B12 (zwitter ionic), Vitamin B2 (uncharged), crystal violet (cationic) and phenol red (anionic) were used as model drugs. Attractive interactions between the negatively charged silicate surfaces and the drug resulted in slower release rates, while repulsive interactions between the two increased the rate of drug elution [58]. The authors highlight the mucoadhesive properties of their hydrogels that increased the efficiency of drug delivery [58]. A similar study by Takahashi *et al*. described the ability of a PEO-polyamide blockcopolymer-Laponite nanocomposite to deliver an uncharged hydrophobic model drug, pyrene [59]. The molecular interactions between the Laponite and the pyrene resulted in sustained release drug delivery profiles to a period of weeks [59].

## 5. Polymer Bioactive Glass Nanocomposites for Tissue Engineering and Repair

Previous and recent research suggest that degradable polymers in combination with bioactive nanostructured materials containing silicon dioxide (e.g., silicate, Bioglass, wollastonite, silicon-doped calcium phosphate) exhibit excellent bioactivity and promote apatite formation *in vitro* and *in vivo* [60].

**Figure 4:**
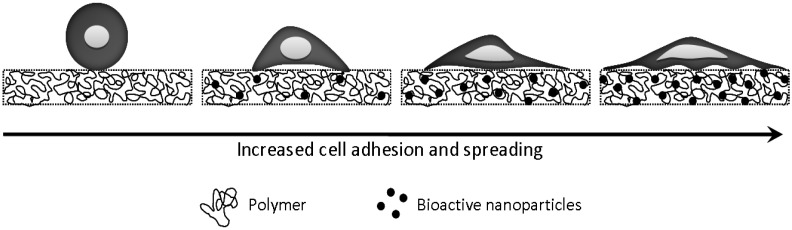
Adherent cells often do not attach to unmodified polymers, exhibiting an unnatural spherical morphology. However, incorporation of bioactive nanoparticles, such as bioglass, enables a more natural cell morphology.

Early studies showed that silicon normally present *in vivo* is essential in the formation of cartilage and bone by participating in cell metabolism [61,62,63,64]. For example, studies by Schwarz *et al.* [63,64]. suggested that silica may act as a cross-linking agent in connective tissue, and Hensch *et al*. investigated the interactions of living tissue with bioactive glasses [62]. Such bioactive glasses were further developed by Hench *et al*. to repair bone defects and influence cell growth (Figure 4) [65]. Vogel *et al.* further investigated how the solubility of bioactive glass affects bond formation between bone and implants. Implantation studies of bioactive glasses in rabbits found these materials to be nontoxic and non inflammatory [66]. Overall the properties of bioactive glass ceramics have been studied well [67]. Many studies investigating implants containing bioactive silicate found that the implants induce bone formation, stimulate osteogenic proliferation and activate bone-related gene expression [68,69]. A desirable feature these implants offer is the capability to promote bone tissue formation at their surface and bond to the surrounding tissue, thus allowing implant fixation for hard tissue engineering applications [70,71].

Some of the polymer bioglass composite materials were found to be suitable for both hard and soft tissue engineering. For example, Blaker *et al.* investigated poly(DL-lactic acid) (PDLLA) Bioglass composites for bone tissue engineering [72]. Meanwhile, Verrier *et al.* showed that porous foams from PDLLA Bioglass support and positively influence the growth and cell behavior of human osteosarcoma cells and human lung carcinoma cells, thus opening avenues to lung tissue engineering applications [73]. Studies of Day *et al.* have investigated the morphology of fibroblasts and observed neovascularization into bioglass coated polyglycolic acid meshes [74]. While these three studies used micron sized bioglass particles, nanosize particles may work as well.

Compared to conventional ceramic materials (grain sizes greater 100 nm), nanophase or nanoparticle containing ceramic materials (grain sizes smaller 100 nm) enhance osteoblast adhesion, proliferation and increase the synthesis of alkaline phosphatase and mineralization [75]. On their own, bioactive glasses have weak mechanical properties which restricts their use for applications requiring strength (such as orthopaedic surgery). Hence a promising approach is the combination of bioactive glasses with polymers to add mechanical strength and toughness [71]. Thus polymer nanocomposites containing bioactive silicate nanoparticles may open new possibilities and strategies in the field of bone repair and dentistry. Several approaches in this direction have been reported (Table 4).

**Table 4:** Polymer-bioactive glass nanocomposites.

Nanoparticles	Polymer	Experimental observations	Ref
Bioglass	P3HB	Nanocomposite supported osteoblast cell attachment, proliferation and differentiation.	[76]
Bioglass	P3HB	Addition of nanoparticles enhanced modulus and strength of the nanocomposite compared to microcomposite. Addition of bioglass resulted in deposition of hydroxyapatite when submersed in simulated body fluid.	[77]
Wollastonite	PCL	Addition of wollastonite improved the nanocomposite Young’s modulus, tensile strength and fracture toughness. Nanocomposites supported *in vitro* formation of apatite.	[78,79]
Bioglass	PLA	Addition of bioglass fiber enhanced *in vitro* bioactivity of the nanocomposite. Significant increase in alkaline phosphatase activity observed in nanocomposite compared to pure PLA.	[80]
Bioglass	Poly L-lactide	Increase in bioglass concentration reduced water absorption capacity but enhanced degradation rate.	[81]
Bioglass	Chitosan & Chitosan-Gelatin	Bioactive nanocomposite scaffolds promoted osteoblast cell adhesion and spreading.	[82,83,84]
Silica	Chitosan	Improved mechanical properties observed due to addition of bioglass. Bioglass aided in significant increase in cell adhesion, proliferation and alkaline phosphatase activity. Enhanced bone regeneration observed when the nanocomposite was implanted *in vivo.*	[85]
Silica	Collagen	Improved bioactivity of the material; accelerated the formation of bone-like apatite and led to the differentiation of human monocytes into osteoclast-like cells.	[86,87]
Silica	Chitin	Chitinous organic matrix provided a template for bio-directed deposition of the silicate mineral phase.	[88]
Silica	Silk	High toughness and strength due to deposition of silica.	[89]
Wollastonite	Silk	Wollastonite enhanced both the mechanical strength and bioactivity of the nanocomposites. *In vitro* cell attachment and proliferation were also observed on the nanocomposites.	[90]

In one of these approaches, Misra *et al*. investigated the effect of bioactive glass nanoparticles on the bioactivity, degradation and *in vitro* cytocompatibility of poly(3-hydroxybutyrate) nanocomposites [60,76]. As expected for polymer nanocomposites, the addition of nanoparticles to the polymer increased the mechanical properties (strength, modulus) when compared to the addition of micron size particles [77]. The polymer nanocomposites were found to be highly bioactive as suggested by the formation of hydroxyapatite on the material surfaces. The weight loss and water uptake were found to increase with increasing bioactive glass content, indicating improvements must be made for the long-term stability of this system. Cytocompatibility studies including alkaline phosphatase activity and osteocalcin production using human MG-63 osteoblast-like cells showed that the polymer nanocomposites are suitable for cell attachment, proliferation and differentiation [76].

Another approach by Kotela *et al*. proposed to develop polymer nanocomposites from polycaprolactone (PCL) and wollastonite nanoparticles for bone repair [78]. Wollastonite is a natural calcium silicate with bioactive properties. Addition of small amounts of wollastonite significantly improved the materials Young’s modulus, tensile strength and fracture toughness. The bioactivity of the polymer nanocomposite was confirmed when apatite formation was observed on the wollastonite surfaces. Overall these results are similar to those previously obtained by Wei *et al*. on polycaprolactone calcium silicate nanocomposites [79]. Other research on nanocomposites considered for bone tissue engineering and repair includes work by Kim *et al*. on PLA bioactive glass nanofiber composites [80]. and work by El-Kady on nanocomposites made from poly L-lactide and bioactive glass nanospheres [81].

The combination of natural polymers such as chitin and chitosan with bioactive silicate nanoparticles allows for designing biocompatible, degradable and cost efficient biomaterials. Recent reports by Peter *et al*. described nanocomposite scaffolds prepared by lyophilizing a chitosan solution containing bioactive glass [82]. Scaffold properties such as swelling, degradation and bioactivity could be modulated when using a chitosan solution or a chitosan-gelatin blend instead of a chitin gel. Addition of gelatin to the chitosan bioglass materials might allow for tuning cell attachment, cell growth, migration and differentiation [83]. The authors pointed out that these polymer nanocomposite scaffolds possess the prerequisites to be further developed for tissue engineering applications [82,83,84].

Another report by Lee *et al*. used a sol-gel method to fabricate chitosan–silicate nanocomposite membranes for bone regeneration [85]. The resulting silicate xerogel was found to be uniformly dispersed in the chitosan matrix on the nanoscale. As expected, the addition of silicate resulted in improved mechanical properties of the nanocomposite when compared to pure chitosan. When immersed in simulated body fluid, the chitosan-silicate nanocomposites induced deposition of calcium phosphate minerals, suggesting *in vitro* bioactivity. A significant increase in osteoblast adhesion, proliferation and alkaline phosphatase activity was enhanced by the presence of silicate. Histological results of polymer nanocomposite implantation in a rat calvarium model showed a significant increase in bone regeneration when compared to the pure chitosan. Overall, improvement in mechanical and biological properties were attributed to the addition of bioactive silica nanostructures into the chitosan matrix [85].

Although pure poly(vinyl alcohol) (PVA) has limited biodegradability when compared to modified PEO and PLGA, PVA has been frequently used in drug delivery, dialysis membranes, wound dressings and other biomedical devices [71]. Thus PVA and bioactive glass or a combination of PVA, chitosan and bioactive glass can be used to prepare scaffold materials that include the mechanical strength of synthetic PVA, the hemocompatibility, bactericidal, and biodegradability of natural chitosan polymers and the bioactive properties of silicate [71].

A different strategy by Heinemann *et al*. attempted to mimic the natural processes of biosilicification to fabricate xerogels from silica and collagen under ambient conditions [86,87]. Sol-gel techniques were used to generate new nanocomposite materials by varying the ratios of collagen and silicate. Addition of calcium phosphate cements further improved bioactivity of the material, accelerated the formation of bone-like apatite and led to the differentiation of human monocytes into osteoclast-like cells. A similar study reported on silica-chitin based bio- nanocomposites fabricated from biological glass sponges [88]. The authors showed that the chitinous organic matrix provides a template for bio-directed deposition of the silicate mineral phase, and the resulting structures can be used for tissue engineering of both bone and cartilage.

Self-assembled nanocomposites from fibrous proteins, such as silk, have been investigated by Foo *et al*. who combined silica nanoparticles with chimeric silk proteins [89]. Films and fibers were fabricated from spider silk, and silica nanoparticles were deposited on this polymer using silicification reactions. The morphology and structure of the silica nanoparticles was controlled by formulation, and the mechanical properties, such as toughness and strength, were improved [89]. Silk proteins were also combined with wollastonite to prepare mechanically strong and bioactive scaffolds that support cell growth [90].

## 6. Future Trends and Challenges

The polymer nanocomposite approach has shown the greatest potential in the design of novel polymeric biomaterials with advanced properties and functionalities. The growing number of available nanoparticles with controllable size and shape further enables researchers to explore promising polymer nanocomposites with better performance than its pristine polymeric counterparts. Mechanically strong polymer nanocomposites can be used either for hard tissue replacement, such as bone, or soft tissue repair like cartilage or tendon. On the other hand, polymer nanocomposites that show responsiveness to external stimuli can direct the design of biomedical devices for better spatial and timely control.

Like other newly arising disciplines, the polymer nanocomposite biomaterials area provides both opportunities and challenges. The lack of well known structure-property relationships between polymer and nanoparticle hampers the design of complex biomedically useful materials. The available database for these materials does not give a well-established theory to predict the properties resulting from the combination of nanoparticles and polymeric biomaterials [91]. We also do not understand to what extent the current composite theory can apply to polymer nanocomposites [92]. From an applications point of view, how can we apply these novel properties to design a medical device? Can these novel properties intimately integrate with currently used medical devices?

The biocompatibilities of polymer nanocomposites also must be taken into account. Although most studies use biocompatible polymers to prepare nanocomposites, the biocompatibility of polymers do not directly apply to polymer nanocomposites. The *in vitro* results of nanoparticle cytotoxicity studies show ambiguities among different research labs or methods. How tissues or the immune system react with polymer nanocomposites is further confounded with the superposition of the different biological properties of nanoparticles and polymers. This is further complicated by the deficiency of knowledge about the *in vivo* fate of nanoparticles.

These issues and questions suggest the polymer nanocomposite approach to design biomaterials is still in its infancy. Although the analysis of chemical, physical and biological properties of polymeric nanocomposite biomaterials seems to be challenging, the multifaceted properties of polymer nanocomposites also provide opportunities to mimic Nature’s expertise in producing materials with excellent performance. Undertaking these challenges can elucidate more details and understanding regarding how polymeric biomaterials and nanoparticles work together.

Overall, this literature review suggests that only few groups are working on developing polymer- silicate (clay) nanocomposites for biomedical applications such as tissue engineering and drug delivery. Most of this research increases our fundamental understanding of materials properties. Research published on polymers in combination with bioglass derived nanostructures and nanoparticles seem to generate more interest within the research communities as these nanocomposites have immediate biomedical relevance and many of them are made of starting materials that are well known (and FDA approved). Nevertheless, preliminary results are promising, and further investigations may help to better understand cell-polymer nanocomposite interactions, immunological reactions and *in vivo* responses.

## Abbreviations

MMT: Montmorillonite clay, layered silicate
P3HB: Poly(3-hydroxybutyrate)
PCL: Polycaprolactone
PEG: Poly(ethylene glycol)
PEO: Poly(ethylene oxide)
PNIPAM: Poly(N-isopropylacrylamide)
PLA: Poly lactic acid
PLG: Poly(lactic–Cco-glycolide)
PLLA: Poly L-lactic acid
